# Case Report: Pediatric respiratory viral infection failure: a case series of eight fatalities in children under 5 years old in Iran

**DOI:** 10.3389/fped.2025.1396142

**Published:** 2025-05-09

**Authors:** Mahnaz Ramzali, Saeed Samadizadeh, Mohsen Ebrahimi, Leila Barati, Britt Nakstad, Alireza Tahamtan

**Affiliations:** ^1^ Department of Microbiology, Faculty of Medicine, Golestan University of Medical Sciences, Gorgan, Iran; ^2^ Wellcome-Wolfson Institute for Experimental Medicine, Queen’s University Belfast, Belfast, United Kingdom; ^3^ Neonatal and Children’s Hospital Research Centre, Golestan University of Medical Sciences, Gorgan, Iran; ^4^ Department of Pediatric and Adolescent Health, University of Botswana, Gaborone, Botswana; ^5^ Division of Paediatric and Adolescent Medicine, Institute of Clinical Medicine, University of Oslo, Oslo, Norway; ^6^ Infectious Diseases Research Center, Golestan University of Medical Sciences, Gorgan, Iran

**Keywords:** case series, viral respiratory infections, pediatric failure, acute respiratory tract infection, Iran

## Abstract

Acute respiratory tract infection (ARTI) remains a major health threat to children under five, contributing to significant morbidity and mortality worldwide. According to the World Health Organization (WHO), ARTI leads to the hospitalization of over 12 million children annually, with approximately one million fatalities, one-third of which occur in low-income countries. The respiratory tract hosts diverse microorganisms, among which pathogenic viruses and bacteria are the leading causes of ARTI. Several risk factors—including age, gender, living conditions, seasonality, and underlying diseases—can influence disease severity. Documenting detailed case series that highlight the clinical characteristics and outcomes of pediatric ARTI, particularly in children with complex underlying conditions, is essential for understanding its impact and guiding clinical decision-making. This manuscript presents a case series of eight pediatric patients from Taleghani Children's Hospital in Gorgan, northern Iran, who tragically succumbed to respiratory viral infections, offering insights into the challenges of managing severe ARTI in children.

## Introduction

Acute respiratory tract infection (ARTI) is a significant health concern for children under five, causing illness and deaths worldwide (1). According to WHO, more than 12 million children are hospitalized annually due to ARTI, and around one million die. A third of the fatalities occur in low-income countries (2–4). The respiratory tract hosts a variety of microorganisms, with pathogenic respiratory viruses and bacteria being the primary causes of ARTI (5, 6). The most prevalent viruses linked to ARTIs are respiratory syncytial virus (RSV), human metapneumovirus (HMPV), influenza viruses, human parainfluenza viruses (HPIVs), human rhinovirus (HRV), and human coronaviruses (HCoVs) (7). Among them, RSV is widespread and can cause a range of clinical symptoms and manifestations, including life-threatening illnesses, particularly in high-risk cases such as infants with chronic lung disease or congenital heart disease, prematurity, and children with weakened immune systems (8).

Multiple risk factors, such as age, gender, living environment, seasonality, and underlying diseases, can significantly affect the severity of the disease (9). Publishing detailed case series, including characteristics and outcomes of ARTI in children, particularly those with complex underlying diseases, is important for describing the impact and outcomes of ARTI among children and assisting clinicians in managing complicated cases. Here, we report a case series of eight pediatric patients from Taleghani Children's Hospital in Gorgan, northern Iran, who tragically succumbed to viral respiratory infections. As all cases presented symptoms suspected to the viral respiratory infections, so the cases have been tested for respiratory viruses. This showed that infections other than RSV, such as HMPV, HRV, HPIV, and SARS-CoV-2, can lead to severe respiratory failure and fatalities in children with underlying health conditions.

## Case 1

A 7-month-old boy was referred with severe respiratory distress symptoms, needing an oxygen hood. This patient was diagnosed with underlying diseases such as spinal muscular atrophy (SMA) and chronic heart disease, along with a two-month history of weight loss, feeding difficulties, and swallowing challenges. Upon admission, clinical pneumonia was diagnosed based on symptoms such as fever, cough, chest pain and weakness. His declining blood oxygen saturation (SpO_2_ = 90%) necessitated his transfer to the pediatric intensive care unit (PICU). On the second day, bilateral infiltration was diagnosed, after which methylprednisolone was administered, and fluoxetine was initiated for depression and panic attacks. The test results of SARS-CoV-2 and influenza were negative. Four days later, he experienced renewed respiratory distress, with SpO_2_ dropping to 95% and presenting a mild fever. Consequently, he was placed on a ventilator. The patient was treated with ceftriaxone, meropenem, and fluoxetine spray to reduce inflammation and mucus production in the lungs and improve breathing and oxygenation, along with 7% saline to help clear mucus and sputum from the airways. The use of fluoxetine was based on the infant's severe irritability and panic-like episodes, which were difficult to diagnose in such a young child. While fluoxetine is not FDA-approved for infants, it was prescribed off-label due to the absence of suitable alternatives and expert clinical judgment. The risks, including potential side effects, were closely monitored, and the decision was made after careful evaluation of the symptoms and treatment needs. On the eighth day of his hospitalization, his symptoms abated, and he regained alertness. Three days later, he developed a fever, and meropenem was continued, and vancomycin was added. His SpO_2_ was now 95%. Two days later, his oxygen levels and hemoglobin (Hb = 8 g/dl) decreased, and he received a blood transfusion. On the 14th day, he deteriorated, needing mask and bag resuscitation and administration of five doses of epinephrine due to bradycardia. Despite undergoing cardiopulmonary resuscitation (CPR), the patient ultimately passed away after a gradual decline in SpO₂, worsening bradycardia, and subsequent cardiac arrest. Of note, the nasopharyngeal swab tested positive for RSV-A. Further information and laboratory findings are shown in Table 1.

**Table 1:** Clinical and laboratory findings of all cases at admission.

Variable	Case 1	Case 2	Case 3	Case 4	Case 5	Case 6	Case 7	Case 8
Age (month)	7	36	7	6	12	11	60	60
Gender	Male	Female	Male	Female	Female	Female	Male	Female
Clinical findings								
Main symptoms	Respiratory distress	Hysteria, Fever, Vomiting	Cough, Tachypnea	Convulsions, Respiratory distress	Respiratory distress	Fever, Respiratory distress	Bloody diarrhea	Lethargy, Fever, Nausea
Underlying disease	SMA, Heart disease	Propionic acid metabolic	SMA	Infantile spasm	Heart disease and emphysema	Down's syndrome	Nephrotic syndrome	Periodic epistaxis
Laboratory findings								
WBC (cells/µl)	7.000	17.900	13.200	5.700	15.500	27.700	8.500	200
RBC (mil/mm3)	5.75	3.34	4.83	5.31	4.59	5.64	5.44	4.22
Hb (g/dl)	15.1	8.5	11.2	15.9	12	16.3	15	10.6
Hct (%)	46.7	29.4	37.2	45.8	39.7	49.5	47.4	33.9
MCV (fl)	81.22	88.02	76.15	86.25	90.43	87.77	87.13	80.33
MCH (pg)	26.6	25.45	21.79	29.94	26.42	28.9	27.29	25.12
MCHC (g/dl)	33.23	28.91	28.61	34.72	29.22	32.93	31.3	31.27
PLT	258.000	257.000	298.000	58.000	97.000	64.000	327.000	16.000
PMN	57	63	72	85	72	73	85	21
Lymph	38	34	28	15	24	24	15	77
BUN (mg/dl)	7	11	7	26	38	35	53	59
Creatinine (mg/dl)	0.5	0.5	0.5	0.7	0.7	1.2	7.7	1.06
Na (mg/dl)	137	136	140	145	145	134	141	152
K (mg/dl)	4.2	5.2	5.4	4	2.16	5.8	5.4	2.04
CPK	N	N	476	N	147	1,921	N	2,449
ESR (mm/hr)	7	10	11	8	40	28	26	N
LDH	N	N	756	N	N	8,622	N	1,164
AST	39	66	48	115	N	2,570	N	213
ALT	7	47	43	91	N	1,094	N	112
CSF analysis								
Glucose	67	N	N	138	N	79	N	N
Protein	27	N	N	51	N	20	N	N
WBC	1	N	N	1	N	1	N	N
Arterial blood gas (ABG) test								
PO2	97	123	41	64	33	108	127.3	N
PCO2	34.1	22.5	55	65.1	54	28.7	31.1	N
HCO3	20.6	15.9	N	N	43.7	N	13.7	N

SMA, spinal muscular atrophy; WBC, white blood cell; RBC, red blood cell; Hb, hemoglobin; Hct, hematocrit; MCV, mean corpuscular volume; MCH, mean corpuscular hemoglobin; MCHC, mean corpuscular hemoglobin concentration; PLT, platelet count; PMN, polymorphonuclear neutrophils; BUN, blood urea nitrogen; CPK, creatine phosphokinase; ESR, erythrocyte sedimentation rate; LDH, lactate dehydrogenase; AST, aspartate aminotransferase; ALT, alanine aminotransferase; CSF, cerebrospinal fluid, PO2, partial pressure of oxygen; PCO2, partial pressure of carbon dioxide; HCO3, bicarbonate; N, not performed.

## Case 2

A 3-year-old girl with propionic acid metabolic disease and speech problems was admitted with symptoms of convulsions, bilateral eyelid twitching, and decreased level of consciousness. This admission occurred after a previous hospital stay nine days earlier, during which she exhibited symptoms including fever and diminished appetite. At admission, the patient experienced increasing upper limb tremors, unresponsive pupils, fever, and frequent vomiting, which was controlled by subsequently prescribing phenobarbital, phenytoin, and levetiracetam during the second to third days of hospitalization. She then experienced frequent seizures despite continuous midazolam infusion for 5 days. On the fourth day, the patient developed respiratory distress with oxygen desaturation. Her respiratory rate increased (at 35 breaths per minute) with severe recessions, and the arterial blood gas analysis revealed a diagnosis of severe metabolic acidosis (values for HCO_3−_ 4.2 mEq/L, PH 7.07, BE −26 mEq/L, PCO_2_ 14.6 mmHg). Bicarbonate infusion was administered, and the O_2_ saturation increased to 98%. A test for SARS-CoV-2 came back positive, and treatment with ceftazidime was continued. The following day, a central venous catheter was placed, and the patient began treatment with metronidazole and vancomycin, along with a packed red blood cell transfusion. Seizures and decreased level of consciousness continued during the next 4 days. On the 10th day of hospitalization, the patient received a regimen that included fresh frozen plasma, vitamin K, continued meropenem, amikacin, ceftriaxone, and a dopamine infusion to prevent complications and secondary infections. On the next day, the patient's general condition did not change, and she was intubated. The next day, the patient had bleeding from the mouth, and fresh frozen plasma was prescribed. On the 15th day of the hospital stay, the patient was severely bradycardic (at 34 breaths per minute), and CPR was initiated. After 45 min, resuscitation was stopped due to a lack of return of spontaneous circulation (ROSC).

## Case 3

A 7-month-old boy with an underlying condition of SMA was admitted to the hospital due to symptoms of shortness of breath, cough, and rapid breathing that had persisted for the past four days without outpatient treatment response. The child had received vaccinations up to the age of two months but had displayed unfavourable growth and developmental patterns. On the first day of hospitalization, ceftriaxone, vancomycin, clindamycin and dexamethasone were provided to treat bacterial infection and reduce inflammation. On the second day, the SpO_2_ decreased to 84%, and he was transferred to PICU, where oxygen supplementation via an oxygen hood resulted in an increase in oxygen saturation to 95%. Clindamycin and cefotaxime treatments were continued. The following day, productive coughs developed as well as bradycardia [Respiratory Rate (RR) = 60, Puls Rate (PR) = 170, and temperature (Tm) = 37]. CPR with cardiac massage was performed, and the patient was revived. On the seventh hospital day, due to further deterioration, the patient's treatment plan was adjusted to include clindamycin and an antiviral neuraminidase inhibitor (Tamiflu). Within a few days, the patient was still breathing with the help of an oxygen hood. On the 18th hospital day, a culture from the endotracheal tube (ETT) returned positive for Pseudomonas aeruginosa, which was sensitive to ciprofloxacin, prompting a resumption of treatment with ciprofloxacin and meropenem, and amikacin and ciprofloxacin was stopped. Ten days later, the patient's condition severely deteriorated, mask-bag ventilation was ineffective, a tracheostomy was placed, and the patient received another transfusion of red blood cells. Notably, the nasopharyngeal swab became positive for HMPV. Four days later, the patient died due to pneumonia, hospital-acquired infection, and the underlying SMA disease.

## Case 4

A 6-month-old female was admitted with convulsions and respiratory distress; fever and sepsis were suspected. The patient had a history of infantile spasms three weeks before admission and episodes of staring and eye rotation 5–6 times daily, worsening in frequency since age two months. At age 4 months, the patient was hospitalized because growth and developmental milestones were delayed and routine vaccinations were incomplete. Upon admission, phenobarbital and levetiracetam were prescribed to control seizures. She experienced fever, runny nose, and decreasing SpO_2_ which improved to 90% with oxygen supplementation. On the second day of hospitalization, her condition worsened, with significant respiratory distress and a decrease in oxygen saturation. She tested positive for HMPV, HRV and HPIV-1, and anti-inflammatory medication was started (Acetaminophen and Ibuprofen). The patient exhibited significant rectal bleeding, prompting an abdominal ultrasound and computed tomography (CT) scan that showed intestinal obstruction and intussusception. The next day, the patient underwent surgery. Two days postsurgery, infectious secretions were seen on the surgical site, and the culture test was positive for Pseudomonas aeruginosa, so antibiotic therapy with ceftriaxone, vancomycin, and clindamycin was commenced. Then the patient experienced a SpO_2_ decrease, the severity of respiratory symptoms, and cardiac arrest was noted. CPR was unsuccessful, and the patient died due to pneumonia and simultaneous infection of three viruses.

## Case 5

A one-year-old girl was admitted with a history of left congenital emphysema and a right lung cyst. One week prior to admission, the patient experienced severe respiratory distress, and SARS-CoV-2 infection was suspected with no test performed. She was on continuous oxygen therapy for the past four months prior to admission, and shortly before admission, she experienced enhanced cyanosis. She was born prematurely, and her growth was stunted despite being nourished with formula milk. On the day of admission, the patient presented as pale, with tachypnea, cyanosis, and a runny nose. Ipratropium (Etruvent) and cefotaxime spray were provided, but due to a gasping breathing pattern and metabolic acidosis, she was intubated and transferred to the PICU. CPR was performed after a few hours because of poor perfusion with non-measurable blood pressure, so dopamine and midazolam infusions were initiated, as well as transfusion of red blood cells and fresh frozen plasma. In the following hours, SpO_2_ decreased to 65%. She developed a fever (Tm 39℃), and vancomycin was initiated. Her acidotic condition worsened (values for HCO_3−_ 15.2 mEq/L, PH 7.27, PCO_2_ 33.1 mmHg). The next day, meropenem was added as well as epinephrine, to improve perfusion and her haemodynamic condition. On the fourth hospital day, she deteriorated with pneumothorax and air was evacuated with pleural drainage. Then, SpO_2_ declined to 30%. She developed bradycardia followed by cardiac arrest. After admission, respiratory viral test were negative. CPR did not result in ROSC, and she demised.

## Case 6

An 11-month-old girl diagnosed with trisomy 21 was admitted with fever (T = 39.5°C) and respiratory distress with influenza-like sympthoms. She had experienced fever for the past six days and productive coughing for the last two days before admission. She had been provided with acetaminophen to reduce fever for five days before admission. At admission, she was hypoxic (SpO2 = 78, PR = 150, Tm = 39.5), was intubated, put on a ventilator, and vancomycin was provided. The fever and tachypnea persisted, an antiviral neuraminidase inhibitor was added on the second day, and anaemia (transfused with red blood cells), leukocytosis, thrombocytopenia, and elevated lactate dehydrogenase (LDH) levels were noted. Then, bradycardia and asystole developed. Notably, the nasopharyngeal swab became positive for HMPV and HRV. Her respiratory condition deteriorated rapidly, with persistent fever, tachypnea, and hypoxia. The infections exacerbated her symptoms, resulting in bradycardia, asystole, and eventual death despite prolonged resuscitation attempts. During CPR, administration of epinephrine and bicarbonate were done every 3–5 min, but ROSC was not achieved, and she demised.

## Case 7

A 5-year-old boy was admitted with respiratory distress and vomiting. The patient had a history of nephrotic syndrome and underwent dialysis 3 times a week. Ultrafiltration dialysis was performed on the first day of hospitalization and repeated 2 days later. Non-invasive respiratory support and oxygen supplementation were administered due to cardiorespiratory depression. He was persistently hypoxic (SpO2 77%) and, therefore, was put on invasive respiratory support. Pneumothorax was also observed in the requested graphs. Subsequently, midazolam was prescribed to treat respiratory depression. To relieve his hypertension (BP 139/99) and to help relax the blood vessels, a vasodilating drug (hydralazine), an ACE inhibitor (enalapril), and a Ca^2+^ blocker were provided, as well as sodium bicarbonate due to respiratory acidosis (HCO_3−_ 12.2 mEq/L, PH 7.33, PO_2_ 113 mmHg, PCO_2_ 23.4 mmHg). The nasopharyngeal swab became positive for HMPV. The following day, he experienced severe bradycardia, leading to cardiac arrest, and unfortunately, he passed away 45 min after CPR was initiated.

## Case 8

A 5-year-old girl was admitted due to weakness, lethargy, respiratory distress, nausea and vomiting. She was anaemic and thrombocytopenic and was transfused with red blood cells and platelets. Prior to admission, she had the experience of periodic epistaxis that was not satisfactorily investigated, as well as poor growth and development. At age 1.5 years, she had chicken pox and then experienced frequent upper airway infections. At admission, she deteriorated, became unconscious and was transferred to the PICU for further monitoring and treatment. An emergency brain CT revealed herniation and an occipital mass. Dexamethasone and a course of methanol were initiated to relieve intracranial pressure. She required respiratory support and supplementation of oxygen; thus, non-invasive ventilation was initiated. On the second day, she received FFP and platelets due to the drop in haemoglobin (7 g/dl) and platelets (5,000 μl). She developed hypotension (blood pressure 98/54) and received an epinephrine infusion. Subsequently, severe hypoxia (SpO2 60%) occurred the following day. The patient's test results came back positive for HRV and HPIV-2. The infections led to the rapid deterioration of her condition, culminating in severe hypoxia and the need for resuscitation. The following day, her condition deteriorated significantly. CPR was initiated but was unsuccessful, and she passed away.

## Discussion

Acute respiratory infections are a significant health concern for infants and children under five, primarily caused by respiratory viruses such as RSV, HMPV, SARS-CoV-2, HRV, and influenza (10–12). These infections manifest in a range of symptoms, from mild cold-like illness to severe bronchiolitis and pneumonia (10, 13), often necessitating hospitalization (13). WHO estimated that 1.9–2.2 million infants and children die due to respiratory infections every year worldwide (14). The severity of the infection can vary depending on the specific virus, the child's age, and any underlying health conditions (13). Among the various respiratory viruses linked to complications in infants and young children, RSV emerges as the predominant cause of bronchiolitis and pneumonia within the first year of life (15). While most children are infected by age two, it can cause severe lower respiratory tract infections, especially in infants (16). HMPV infections often present with similar symptoms to RSV, including cough, nasal congestion, and wheezing, and can also lead to pneumonia (17). It is an important cause of lower respiratory tract infections in children (18). While many children experience mild illness, HMPV can cause severe disease, especially in premature infants and children with underlying health conditions (19). HRVs is a common cause of the common cold and upper respiratory tract infections (12). While typically mild, it can sometimes lead to lower respiratory tract infections, especially in infants and young children (18). Influenza can cause a range of respiratory illnesses, from mild to severe (10). While generally less severe than RSV in very young children, influenza can still lead to serious complications like pneumonia (18).

The COVID-19 pandemic has ushered in profound shifts in human behaviour and society, which has influenced the incidence of non-SARS-CoV-2 respiratory viruses (20). Recent studies indicate that public health measures implemented during the COVID-19 pandemic significantly impacted the circulation patterns of these viruses, leading to altered seasonal distributions and potentially affecting long-term immunity patterns in vulnerable populations (21). Despite the diversity of pathogens associated with acute respiratory infections, the clinical presentation often appears strikingly similar across cases (14). Therefore, the precise identification of causative factors becomes paramount in guiding effective treatment strategies. The purpose of this study is to investigate the causes of respiratory failure in children under 5 years old who have presented with respiratory symptoms. The study aims to identify the underlying causes of respiratory failure in these children, with a focus on viral infections, pneumothorax, and other factors that may contribute to respiratory failure (Table 2). The study also aims to provide insights into the clinical presentation of respiratory failure in children under 5 years old and to identify potential strategies for prevention and treatment.

**Table 2:** Clinical course and outcomes of all cases.

Cases	Initial symptoms at admission	Key diagnostic tests and results	Interventions (e.g., medications, ventilation)	Disease progression and outcome
1	• Severe shortness of breath • Diagnosed with SMA and chronic heart disease • Two months of weight loss and nutritional issues • Admitted due to pneumonia	• Positive for RSV-A	• Methylprednisolone • Fluoxetine for depression • Ventilator support • Ceftriaxone • Meropenem	• Decreased SpO2 • Increasing bradycardia • CPR • Epinephrine • Death after cardiac arrest.
2	• Seizure • Decreased consciousness • History of admission due to fever and decreased appetite • History of metabolic disease	• Positive for SARS-CoV-2 • Severe metabolic acidosis.	• Phenobarbital • Phenytoin • Levetiracetam • Midazolam • Ceftazidime • Metronidazole • Vancomycin • Red blood cell transfusion	• Persistent seizures • Bradycardia • Asystole • CPR initiated and death after 45 min
3	• Shortness of breath • Cough • Rapid breathing • History of SMA	• Positive for HMPV	• Ceftriaxone • Vancomycin • Clindamycin • Dexamethasone • Tamiflu	• Worsening respiratory failure • Need for tracheostomy • Death due to pneumonia and SMA
4	• Seizure • Shortness of breath • Suspected sepsis • History of infantile spasms	• Positive for HMPV, HRV, and HPIV-1	• Phenobarbital • Levetiracetam • Acetaminophen • Ibuprofen • Antibiotics (ceftriaxone, vancomycin, clindamycin) • Bowel obstruction surgery	• Cardiac arrest • Unsuccessful CPR, and death
5	• Severe shortness of breath • History of congenital emphysema and lung cysts	• Suspected SARS-CoV-2 • Respiratory acidosis	• Ipratropium • Cefotaxime • Intubation • Midazolam • Red blood cell transfusion • Epinephrine • Meropenem.	• Pneumothorax • Respiratory failure • Cardiac arrest • Death despite CPR
6	• Fever • Shortness of breath • History of Down syndrome	• Positive for HMPV and HRV	• Vancomycin • Antiviral neuraminidase inhibitors • Red blood cell transfusion • Respiratory support (intubation)	• Tachypnea • Bradycardia • Asystole • Unsuccessful CPR • Death after prolonged resuscitation
7	• Shortness of breath • Vomiting • History of nephrotic syndrome	• Positive for HMPV • Pneumothorax observed	• Dialysis • Non-invasive and invasive respiratory support • Midazolam • Vasodilators • ACE inhibitors • Bicarbonate sodium	• Severe bradycardia • Cardiac arrest • Death after CPR
8	• Weakness • Fatigue • Shortness of breath • Anemia • Thrombocytopenia • History of chickenpox and recurrent airway infections	• Positive for HRV and HPIV-2	• Dexamethasone • Methanol • Red blood cell and platelet transfusion • Non-invasive respiratory support • Epinephrine	• Rapid deterioration • CPR • Death due to severe hypoxia and viral infections

RSV, respiratory syncytial virus; HMPV, human metapneumovirus; HPIVs, human parainfluenza viruses; HRV, human rhinovirus; SARS-CoV-2, severe acute respiratory syndrome-coronavirus 2; SMA, spinal muscular atrophy; CPR, cardiopulmonary resuscitation.

This investigation scrutinized eight pediatric cases of children below 5 who presented with acute respiratory symptoms necessitating hospitalization, culminating in fatalities over 5 months from October 2021 to March 2022 in a northern city in Iran. All cases, including three boys and five girls, had underlying diseases, which serve as risk factors for fatal outcomes due to severe respiratory disease, as outlined in Table 1. According to recent research, gender and age significantly influence the risk and severity of respiratory infections in children under five years. Male infants are notably more susceptible, being 55% more likely to develop severe respiratory infections compared to female infants (22). Regarding age distribution, infants under 11 months bear the highest risk, comprising approximately 83% of RSV-positive cases, while children aged 1–2 years account for 9.2% of infections. The clinical manifestations also vary by age, with bronchiolitis (57.5%) and pneumonia (30.8%) being the most common presentations requiring intensive care unit admission (23). Of note, a 7-month-old boy (case 1) was RSV infected, while another was diagnosed with SARS-CoV-2 (case 2), and one had a suspected SARS-CoV-2 infection a week before admission (case 5). This underlines the significance of diagnosing respiratory viral infections in pediatric patients, in line with previous studies (24, 25). Importantly, HMPV (cases 3, 4, 6, and 7), HRV (cases 4, 6, and 8) and HPIV-1 and 2 infections were diagnosed for cases 4 and 8, respectively. Three cases (cases 4, 6, and 8) had co-infections with two viruses. Co-infections can exacerbate disease severity by increasing viral load, triggering excessive immune responses, and impairing the host's ability to control the infection. Additionally, interactions between viruses could enhance viral replication or suppress immune defenses, further worsening clinical outcomes. Co-infections can exacerbate inflammation in the respiratory tract, potentially leading to increased symptom severity, prolonged illness, and a higher risk of complications like pneumonia (26). The interaction between different viruses can also alter immune responses, making the body more susceptible to secondary bacterial infections (26). Further research is needed to fully understand the complex interplay of viruses in co-infections and their precise effects on the body (27). Also, a clinically suspected infection (case 7) led to pneumothorax, and a history of chickenpox virus infection (case 8) that subsequently experienced multiple episodes of respiratory disease. It seems that many symptom-oriented interventions were being carried out in the cases while the aetiology of the disease remained unclear. So, several of these interventions may exacerbated the already disrupted homeostasis of the body.

Among these patients, information regarding the mode of delivery (vaginal birth or cesarean section) was only available for cases 1, 3, 4, and 7, all of which were delivered by cesarean section, while data on this aspect was insufficient for the remaining cases. Cesarean section may increase the risk of respiratory infections in newborns due to the lack of exposure to the mother's vaginal microbiome, which is crucial for immune system development. Additionally, cesarean-born infants may experience impaired lung fluid clearance, leading to respiratory issues. Studies have shown a higher incidence of respiratory diseases, such as bronchiolitis and asthma, in cesarean-delivered infants compared to those born vaginally (28). Two cases had incomplete routine vaccine coverage, emphasizing the role of vaccination in bolstering the immune system, reducing infection rates, and mitigating symptom severity (29). In accordance with previous papers, being born prematurely influences the occurrence and severity of symptoms (30). Children with underlying diseases, including congenital heart disease (cases 1 and 5), SMA (cases 1 and 3), a history of infantile spasms (case 4), trisomy 21 (case 6), underlying nephrotic syndrome needing regular dialysis (case 7), and metabolic disease (case 2), were all Immunocompromised with an inability to clear respiratory tract infections and therefore at increased risk of fatal outcome.

Overally, a total of 411 infants and young children under five years of age presenting with acute respiratory infections were admitted between October 2021 and March 2022. Among these cases, eight patients passed away. Notably, this sample population was drawn exclusively from the single specialized pediatric hospital serving the metropolitan area. This investigation coincided with the post-COVID-19 pandemic era, which potentially influenced circulating respiratory virus patterns and immune system responses. Our analysis revealed that beyond RSV, various other respiratory viruses, including HMPV, HRV, HPIV, and SARS-CoV-2, contributed significantly to disease severity and mortality in neonatal populations.

## Data Availability

The original contributions presented in the study are included in the article/Supplementary Material, further inquiries can be directed to the corresponding author.
